# 2015/16 seasonal vaccine effectiveness against hospitalisation with influenza A(H1N1)pdm09 and B among elderly people in Europe: results from the I-MOVE+ project

**DOI:** 10.2807/1560-7917.ES.2017.22.30.30580

**Published:** 2017-07-27

**Authors:** Marc Rondy, Amparo Larrauri, Itziar Casado, Valeria Alfonsi, Daniela Pitigoi, Odile Launay, Ritva K Syrjänen, Giedre Gefenaite, Ausenda Machado, Vesna Višekruna Vučina, Judith Krisztina Horváth, Iwona Paradowska-Stankiewicz, Sierk D Marbus, Alin Gherasim, Jorge Alberto Díaz-González, Caterina Rizzo, Alina E Ivanciuc, Florence Galtier, Niina Ikonen, Aukse Mickiene, Veronica Gomez, Sanja Kurečić Filipović, Annamária Ferenczi, Monika R Korcinska, Rianne van Gageldonk-Lafeber, I-MOVE+ hospital working group, Marta Valenciano

**Affiliations:** 1 EpiConcept, Paris, France; 2 National Centre of Epidemiology, Institute of Health Carlos III, Madrid, Spain; 3 CIBER Epidemiología y Salud Pública, Institute of Health Carlos III, Madrid, Spain; 4 Instituto de Salud Pública de Navarra, IdiSNA, Pamplona, Spain; 5 Istituto Superiore di Sanità, Rome, Italy; 6 UMF Carol Davila, INC Cantacuzino, Bucharest, Romania; 7 Inserm, F-CRIN, Innovative clinical research network in vaccinology (I-REIVAC), Paris, France; 8 Université Paris Descartes, Sorbonne Paris Cité, APHP, CIC Cochin-Pasteur, Paris, France; 9 Impact Assessment Unit, National Institute for Health and Welfare, Tampere, Finland; 10 Department of Infectious diseases, Lithuanian University of Health Sciences, Kaunas, Lithuania; 11 Epidemiology Research Unit, Epidemiology Department, National Health Institute Dr Ricardo Jorge, Lisbon, Portugal; 12 Epidemiology Service, Croatian Institute of Public Health, Zagreb, Croatia; 13 Office of the Chief Medical Officer, Budapest, Hungary; 14 National institute of Public Health - National Institute of Hygiene, Department of Epidemiology, Warsaw, Poland; 15 Centre for Epidemiology and surveillance of infectious diseases, Centre for infectious disease control, National Institute for Public Health and the Environment (RIVM), Bilthoven, The Netherlands; 16 INC Cantacuzino, Bucharest, Romania; 17 CIC de Montpellier, Hôpital Saint-Eloi, CHU de Montpellier, Montpellier, France; 18 Viral Infections Unit, National Institute for Health and Welfare, Helsinki, Finland; 19 The I-MOVE+ hospital working group is listed at the end of the article

**Keywords:** influenza, vaccine-preventable diseases, vaccine effectiveness, case control, severe acute respiratory infection, elderly, hospitalisation

## Abstract

We conducted a multicentre test-negative case–control study in 27 hospitals of 11 European countries to measure 2015/16 influenza vaccine effectiveness (IVE) against hospitalised influenza A(H1N1)pdm09 and B among people aged ≥ 65 years. Patients swabbed within 7 days after onset of symptoms compatible with severe acute respiratory infection were included. Information on demographics, vaccination and underlying conditions was collected. Using logistic regression, we measured IVE adjusted for potential confounders. We included 355 influenza A(H1N1)pdm09 cases, 110 influenza B cases, and 1,274 controls. Adjusted IVE against influenza A(H1N1)pdm09 was 42% (95% confidence interval (CI): 22 to 57). It was 59% (95% CI: 23 to 78), 48% (95% CI: 5 to 71), 43% (95% CI: 8 to 65) and 39% (95% CI: 7 to 60) in patients with diabetes mellitus, cancer, lung and heart disease, respectively. Adjusted IVE against influenza B was 52% (95% CI: 24 to 70). It was 62% (95% CI: 5 to 85), 60% (95% CI: 18 to 80) and 36% (95% CI: -23 to 67) in patients with diabetes mellitus, lung and heart disease, respectively. 2015/16 IVE estimates against hospitalised influenza in elderly people was moderate against influenza A(H1N1)pdm09 and B, including among those with diabetes mellitus, cancer, lung or heart diseases.

## Background

Elderly populations, defined as those aged 65 years and above, and, more specifically, elderly people with underlying conditions, are at increased risk for hospitalisation due to influenza [1]. Influenza may also increase the severity of underlying chronic lung diseases, probably through inflammatory processes [2]. Viral pneumonia due to influenza seems to predispose to myocardial infarction, and congestive heart failures are more common during influenza seasons [3]. Patients with cancer treated with chemotherapy [4] and diabetic patients are more vulnerable to influenza. Their impaired immune response [5] could also affect host response to vaccination [6,7]. Evidence of the effectiveness of influenza vaccination in preventing severe clinical outcomes was recently described as low or very low among elderly people [8], and among patients with cancer [9], diabetes mellitus [10], lung diseases [11] [12], or cardiovascular diseases [13].

Despite the Council of the European Union and the World Health Organization’s (WHO) recommendations to annually vaccinate elderly people [14,15], influenza vaccine coverage among elderly people remains below the 75% target in most European countries [16].

In this context, post-marketing studies to estimate the influenza vaccine effectiveness (IVE) among elderly people are needed to inform about vaccination benefits for vaccinees, detect subgroups in which the vaccine performs less well and identify vaccine types that perform best. In 2015, to address this issue, the Integrated Monitoring of Vaccines in Europe plus (I‑MOVE+) consortium initiated a network of hospitals across Europe to measure IVE against laboratory-confirmed hospitalised influenza among elderly people.

The WHO recommended to include in the 2015/16 trivalent influenza vaccine for the northern hemisphere an A/California/7/2009 (H1N1)pdm09-like virus, an A/Switzerland/9715293/2013 (H3N2)-like virus and a B/Phuket/3073/2013-like virus (Yamagata lineage) [17].

In the 2015/16 influenza season in Europe, influenza A(H1N1)pdm09 and influenza B (mainly Victoria lineage) viruses predominated [18]. We conducted a multicentre hospital-based test-negative design (TND) case–control study to measure the 2015/16 seasonal IVE against hospitalisation with influenza A(H1N1)pdm09 and influenza B among elderly people in Europe, by risk groups and for specific vaccine types.

## Methods

### Study sites and design

**Figure 1:**
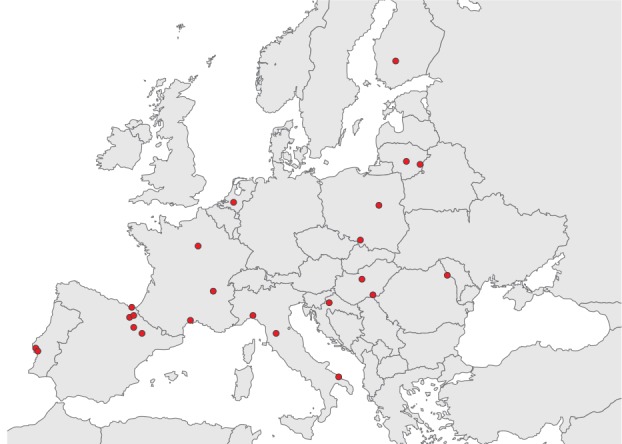
Location of the hospitals participating in the I-MOVE + study, Europe, influenza season 2015/16 (n = 27 hospitals)

We set up a European network of 27 hospitals in 11 countries (Croatia, Finland, France, Hungary, Italy, Lithuania, the Netherlands, Poland, Portugal, Romania and Spain) (Figure 1), organised in 12 study sites (in Spain, Navarre region hospitals had their own coordination centre). Each study site adapted a generic protocol to their local setting [19,20]. Monitoring visits were organised to ensure the study was done similarly across hospitals. We conducted a multicentre TND case–control study.

I MOVE+: Integrated Monitoring of Vaccines in Europe plus.

Each dot represents one location and there may be more than one hospital in one location.

### Study period

In each study site, the study period started at least two weeks after the beginning of the vaccination campaign in the respective countries and lasted from the week of the first detection of a laboratory-confirmed case of influenza to the week of the last laboratory-confirmed case of influenza. We defined different study periods for influenza A(H1N1)pdm09 and B.

### Study population

Our study population included all community dwelling patients aged 65 years and above who had no contraindication for influenza vaccination or previous laboratory-confirmed influenza in the season and agreed to participate. In the participating services of each hospital, patients admitted for clinical conditions that could be related to influenza were screened for eligibility. Study physicians, nurses or collaborating medical staff asked patients about onset of symptoms compatible with the definition of a severe acute respiratory infection (SARI) in the previous 7 days.

We defined a SARI case as a hospitalised patient with at least one systemic (fever or feverishness, malaise, headache, myalgia or deterioration of general or functional condition) and at least one respiratory sign or symptom (cough, sore throat or shortness of breath) at admission, or within 48 hours after admission.

### Data collection

The hospital study teams swabbed patients meeting the SARI case definition. Specimens were tested by RT-PCR and patients classified as influenza A(H1N1)pdm09 cases, influenza B cases, other influenza cases or controls if their specimens tested negative for any influenza virus.

The hospital study teams collected information on patients’ age and sex, influenza vaccination status including date and brand of the 2015/16 vaccine and the status in two previous seasons and underlying conditions listed for clinical risk groups recommended for influenza vaccination [21]. The underlying conditions included diabetes mellitus, obesity (defined as body mass index ≥ 30 kg/m2), cardiovascular conditions (such as congenital heart disease, congestive heart failure and coronary artery disease), lung diseases (such as chronic obstructive pulmonary disease, cystic fibrosis, asthma), renal and rheumatologic diseases, cancer, stroke, dementia and cirrhosis. Information on number of hospitalisations for underlying conditions in the previous 12 months, number of general practitioners (GP) visits in the previous three months, smoking status and functional impairment (based on Barthel index score [22]) was also collected.

Information on demographics and underlying conditions were collected from interviews with patients (or their relatives) and hospital and/or primary care databases. In study sites with no vaccination register, vaccination status was collected through interview with patients. For patients vaccinated or unable to provide their vaccination status, study sites called patients’ GP or pharmacists to retrieve vaccination status, date and brand (Table 1).

**Table 1:** Vaccine types used and source of information for vaccination status by study site, I-MOVE + study, Europe, influenza season, 2015/16

Study site	Number of hospitals	Vaccines used	Data sources	
			Source of information for vaccination status	Source of information for underlying conditions
Croatia	1	Inactivated subunit	I	I; H
Finland	2	Inactivated split	R; I; GP	I; GP; H
France	3	Inactivated subunit; inactivated split	I; P	I; H
Hungary	2	Adjuvanted	I; GP	I; H
Italy	3	Inactivated subunit; inactivated split; adjuvanted	I; GP	I; H
Lithuania	2	Inactivated subunit	I; GP	I; H
Navarre	3	Inactivated split	R	I; GP; H
The Netherlands	1	Inactivated subunit; inactivated split	I	I; H
Poland	3	Unknown	I; GP	I; H
Portugal	2	Inactivated subunit; inactivated split	R; I; GP	I; H
Romania	3	Inactivated subunit	I; GP	I; H
Spain	2	Inactivated subunit; inactivated split	R; I; GP	I; H

GP: general practitioner/primary care database; H: hospital database/medical charts; I: interview with patient; I-MOVE+: Integrated Monitoring of Vaccines in Europe plus; P: pharmacist; R: register.

We defined patients as vaccinated with the 2015/16 influenza vaccine if they had been vaccinated at least 14 days before symptoms onset. Otherwise, they were considered as unvaccinated.

### Data analysis

We computed the IVE as (1 minus the odds ratio (OR) of vaccination between cases and controls) x 100. We performed a pooled one-stage analysis using the study site as a fixed effect and estimated IVE stratified on the presence of underlying conditions. All IVE estimates were adjusted for study site, date of SARI symptom onset and age modelled as restricted cubic splines with four knots (initial model). To adjust for additional potential confounders (sex, each group of underlying conditions, hospitalisation in the past year, more than one GP visit in the past 3 months, functional impairment, current smoking), we performed a multivariable analysis using an onward step by step modelling and analysing them as dichotomous variables. Patients with missing covariates were excluded from the analyses adjusted for these covariates. We retained in the model (full model) all covariates that changed the IVE estimate by 10% of more (relative change).

We grouped the vaccine brands in split virion, subunit or adjuvanted vaccines. To compute vaccine type-specific effectiveness, we restricted our analyses to countries with at least one patient vaccinated with a specific type.

We also computed a pooled IVE with a two-stage model, adjusting study site-specific IVE for study site-specific confounders (same as listed above) when sample size allowed. We quantified the heterogeneity between site estimates using the I-square [23].

To minimise the inclusion of false influenza-negatives in the control group, we carried out sensitivity analyses by restricting population to (i) patients swabbed up to three days after symptom onset and (ii) patients not treated with antivirals until the day before swabbing.

## Results

A total of 2,077 patients meeting the inclusion criteria were recruited in the study. We excluded 472 controls (23%) recruited outside of the study period and 65 patients (4%) with missing information on vaccination status. We included 1,274 controls and 528 cases, of which 353 (67%) were influenza A(H1N1)pdm09 positive, 105 (20%) were influenza B-positive, 41 (8%) were influenza A(H3N2)-positive, 24 (5%) were influenza A (non-subtyped)-positive, two (<1%) were co-infected by influenza A(H1N1)pdm09 and B, one (<1%) was co-infected by influenza A(H3N2) and B and two (<1%) were co-infected by influenza A (non-subtyped) and B. Of the 52 cases of influenza B with a known lineage, 47 (90%) were Victoria and 5 (10%) were Yamagata. The 42 cases positive for influenza A(H3N2) did not allow us to compute IVE against this subtype.

**Figure 2:**
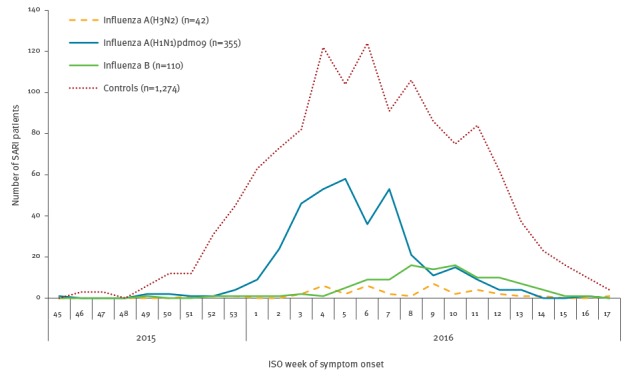
Cases of severe acute respiratory infection with influenza A(H3N2), A(H1N1)pdm09, and B, and negative controls, I-MOVE+ study, Europe, influenza season 2015/16 (n = 504 cases^a^; n = 1,274 controls)

The maximum number of influenza A(H1N1)pdm09 cases were recruited in weeks 5 to 8 of 2016 and the maximum number of influenza B and A(H3N2) cases in week 10 (Figure 2).

I-MOVE+: Integrated Monitoring of Vaccines in Europe plus; ISO: International Organisation for Standardisation; SARI: severe acute respiratory infection.

^a^ Including two influenza A(H1N1)pdm09 and B co-infections and one influenza A(H3N2) and B co-infection.

Overall, 216/528 cases (41%) and 694/1,274 controls (54%) had received trivalent inactivated vaccines. Among those vaccinated, 51 (6%) received adjuvanted vaccines, 338 (37%) inactivated subunit vaccines,513 (56%) inactivated split virion vaccines and the information on vaccine type was missing for 8 (1%) vaccinated patients. Age and time adjusted IVE against any influenza was 39% (95 % confidence interval (CI): 22 to 53).

### Vaccine effectiveness against hospitalised influenza A(H1N1)pdm09

We included in this analysis 355 cases of influenza A(H1N1)pdm09, of whom 138 (39%) were vaccinated, and 976 controls, of whom 543 (56%) vaccinated. The median age of A(H1N1)pdm09 cases and controls was 76 (Interquartile range (IQR) = 12 years) and 78 (IQR = 12 years) years respectively (p = 0.001). The proportion of patients with underlying conditions was similar among cases and controls except for renal (16% among cases vs 23% among controls, p = 0.003) and rheumatologic diseases (6% among cases vs 11% among controls, p = 0.033). Ten percent of A(H1N1)pdm09 cases and 3% of controls had received antivirals before swabbing (p < 0.001) and 61% of cases vs 53% of controls were swabbed within 3 days after symptoms onset (p = 0.013) (Table 2).

**Table 2:** Characteristics of influenza A(H1N1)pdm09 and influenza B hospitalised cases and corresponding test-negative controls included in the I-MOVE +  study, Europe, influenza season 2015/16

	Influenza A(H1N1)pdm09				Influenza B			
	Cases (n = 355)		Controls (n = 976)		Cases (n = 110)		Controls (n = 1,015)	
	n	%	n	%	n	%	n	%
Median age in years (range)	76 (65–95)	–	78 (65–101)	–	76 (65–94)	–	78 (65–101)	–
Aged 65–79 years	235/355	66.2	535/976	54.8^a^	76/110	69.1	566/1,015	55.8^a^
Sex = male	194/351	55.3	512/975	52.5	57/110	51.8	520/1,014	51.3
2015/16 seasonal influenza vaccination	138/355	38.9	543/976	55.6^a^	50/110	45.5	588/1,015	57.9^a^
2014/15 seasonal influenza vaccination	136/347	39.2	537/958	56.1^a^	53/109	48.6	589/998	59.0^a^
**Type of vaccine**								
Not vaccinated	217/353	61.5	433/970	44.6^a^	60/110	54.5	427/1012	42.2^a^
Inactivated subunit	77/353	21.8	209/970	21.5	20/110	18.2	207/1012	20.5
Inactivated split virion	59/353	16.7	312/970	32.2	30/110	27.3	332/1012	32.8
Adjuvanted	0/353	0.0	16/970	1.6	0/110	0.0	46/1012	4.5
**Underlying conditions**								
Diabetes	99/347	28.5	277/954	29.0	31/104	29.8	284/992	28.6
Heart disease	215/351	61.3	590/967	61.0	63/107	58.9	631/1,006	62.7
Lung disease	141/351	40.2	438/965	45.4	46/108	42.6	484/996	48.6
Immunodeficiency	7/343	2.0	34/942	3.6	10/106	9.4	32/986	3.2^a^
Cancer	93/350	26.6	263/963	27.3	19/105	18.1	280/1,001	28.0^a^
Nutritional deficiency	16/239	6.7	65/723	9.0	9/84	10.7	51/753	6.8
Renal disease	54/349	15.5	221/960	23.0^a^	20/106	18.9	236/996	23.7
Dementia or stroke	46/346	13.3	160/956	16.7	17/104	16.3	156/991	15.7
Rheumatologic disease	15/246	6.1	80/737	10.9^a^	11/87	12.6	83/757	11.0
Obesity^b^	43/349	12.3	139/951	14.6	5/104	4.8	123/985	12.5^a^
Any underlying condition	325/350	92.9	908/976	93.0	99/110	90.0	955/1,015	94.1
≥ 2 underlying conditions	244/347	70.3	719/964	74.6	72/108	66.7	752/1,006	74.8
Functional impairment^c^	116/347	33.4	347/948	36.6	20/109	18.3	359/988	36.3^a^
Hospitalisation in past 12 months	152/345	44.1	446/960	46.5	39/108	36.1	475/989	48.0^a^
Current smoking	79/340	23.2	183/901	20.3	36/102	35.3	210/927	22.7^a^
**Study sites**								
Croatia	16/355	4.5	15/976	1.5	5/110	4.5	3/1,015	0.3
Finland	18/355	5.1	57/976	5.8	3/110	2.7	35/1,015	3.4
France	11/355	3.1	124/976	12.7	26/110	23.6	124/1,015	12.2
Hungary	0/355	0.0	0/976	0.0	1/110	0.9	5/1,015	0.5
Italy	3/355	0.8	102/976	10.5	10/110	9.1	249/1,015	24.5
Lithuania	17/355	4.8	41/976	4.2	3/110	2.7	31/1,015	3.1
Navarra	87/355	24.5	240/976	24.6	27/110	24.5	230/1,015	22.7
The Netherlands	5/355	1.4	12/976	1.2	3/110	2.7	6/1,015	0.6
Poland	17/355	4.8)	14/976	1.4	6/110	5.5	9/1,015	0.9
Portugal	14/355	3.9	35/976	3.6	1/110	0.9	1/1,015	0.1
Romania	58/355	16.3	101/976	10.3	2/110	1.8	70/1,015	6.9
Spain	109/355	30.7	235/976	24.1	23/110	20.9	252/1,015	24.8
**Potential for misclassification**								
Antivirals before swabbing	36/353	10.2	32/972	3.3^a^	7/107	7.5	27/1,012	2.7^a^
Swabbing within 3 days of onset	216/355	60.8	518/976	53.1^a^	54/110	49.1	585/1,015	57.6

I MOVE+: Integrated Monitoring of Vaccines in Europe plus.

^a^ Indicates a significant difference (p value < 0.05) between cases and controls.

^b^ Defined as body-mass index ≥ 30 kg/m2.

^c^ Defined as Barthel score < 100 [22].

One-stage pooled IVE against A(H1N1)pdm09 adjusted for onset time and age was 42% (95% CI: 22 to 57) and 39% (95% CI: 17 to 56) when further adjusted for a range of underlying conditions and hospitalisation in the previous year (Table 3). IVE against A(H1N1)pdm09 was 59% (95% CI: 23 to 78), 48% (95% CI: 5 to 71), 43% (95% CI: 8 to 65) and 39% (95% CI: 7 to 60) in patients with diabetes mellitus (n = 362), cancer (n = 346), lung (n = 573) and heart disease (n = 792), respectively (Table 3).

**Table 3:** Pooled adjusted seasonal influenza vaccine effectiveness against hospitalised influenza A(H1N1)pdm09 overall among elderly people, by risk groups and vaccine type, I-MOVE +  study, Europe, influenza season, 2015/16

Analyses	Model used for adjustment^a^	Vaccinated /cases	Vaccinated /controls	Adjusted IVE	95% CI
**Overall**					
	Initial	138/355	543/976	42.4	22.0 to 57.4
	Full	131/336	509/923	39.4	16.6 to 55.9
**By risk groups**					
At least one underlying condition	Initial	130/317	499/892	35.7	11.4 to 53.3
	Initial plus severity			35.6	11.2 to 53.3
Diabetes mellitus					
No	Initial	98/242	370/674	33.9	4.6 to 54.2
Yes	Initial	33/96	150/266	58.5	22.8 to 77.7
	Initial plus severity			58.5	22.7 to 77.8
Heart disease					
No	Initial	54/131	207/372	37.3	-1.2 to 61.1
Yes	Initial	80/211	321/581	38.4	6.5 to 59.5
	Initial plus severity			39.0	7.3 to 59.9
Lung disease					
No	Initial	61/203	250/515	39.7	8.0 to 60.4
Yes	Initial	72/139	276/434	42.4	7.2 to 64.3
	Initial plus severity			42.8	7.8 to 64.5
Cancer					
No	Initial	93/252	375/691	35.7	6.7 to 55.7
Yes	Initial	41/90	150/256	47.7	4.8 to 71.3
	Initial plus severity			47.8	4.8 to 71.4
**Vaccine type**					
Inactivated subunit	Initial	77/224	209/538	28.1	-8.6 to 52.4
Inactivated split virion	Initial	59/178	312/588	54.7	30.7 to 70.4
**Sensitivity analyses**					
Two-stage model	two-stage^b^	132/329	527/932	49.0	13.5 to 70.0
Restricted to patients swabbed within 3 days	Initial	85/216	313/518	49.1	23.8 to 66.0
Restricted to patients not receiving antivirals before swabbing	Initial	126/317	531/940	42.2	20.8 to 57.8

CI: confidence interval; I MOVE+: Integrated Monitoring of Vaccines in Europe plus; IVE: influenza vaccine effectiveness.

^a^ Initial: one-stage model adjusted for study site, date of symptom onset and age (modelled as restricted cubic splines). Full: one-stage model adjusted for study site, date of symptom onset, age (modelled as restricted cubic splines), lung, heart, renal disease, diabetes mellitus, cancer, obesity (body-mass index ≥ 30 kg/m^2^) and hospitalisation for underlying conditions in past year. Severity: hospitalisations for underlying conditions in the previous year.

^b^ Poland and Hungary were excluded because there were no vaccinated controls in Poland and no cases in Hungary.

IVE against A(H1N1)pdm09 was 28% (95% CI: −9 to 52) for inactivated subunit vaccines (n = 762) and 55% (95% CI: 31 to 70) for inactivated split virion vaccines (n = 716).

Study site specific IVE ranged between −152% (95% CI: −3,081 to 80) in Italy (n = 105) and 95% (95% CI: 7 to 100) in the Netherlands (n = 17) (Table 4). The statistical heterogeneity between study site specific IVE estimates was moderate (I2 = 36%). The two-stage pooled analysis (n = 1,261) included Croatia, Finland, France, Italy, Lithuania, Navarre, the Netherlands, Portugal, Romania and Spain. IVE was 49% (95% CI: 14 to 70). In sensitivity analyses, IVE against influenza A(H1N1)pdm09 was 42% (95% CI: 21 to 58) when restricting to patients not having received antiviral treatment (n = 1,257) and 49% (95% CI: 24 to 66) among patients swabbed within 3 days of symptoms onset (n = 734) (Table 3).

**Table 4:** Study site specific and two-stage^a^ pooled seasonal vaccine effectiveness against hospitalised influenza A(H1N1)pdm09 among elderly people, I- MOVE + study, Europe, influenza season 2015/16 (n = 1,261)

Study site	Inclusion period	Variables used for adjustment^b^	Vaccinated /cases	Vaccinated /controls	Adjusted IVE	95% CI	I-square
Croatia	2016w5–2016w13	Date	4/16	1/15	-122.0	−4,314.5 to 88.8	–
Finland	2015w50–2016w7	Date	5/18	38/57	85.0	43.7 to 96.0	–
France	2016w4–2016w14	Date	3/11	84/124	83.7	32.2 to 96.1	–
Italy	2016w5–2016w11	Date	2/3	47/102	-152.2	−3,081.1 to 80.0	–
Lithuania	2016w2–2016w10	Date	1/17	7/41	66.8	−210.4 to 96.4	–
Navarra	2015w46–2016w13	Date	46/87	169/240	45.9	5.3 to 69.1	–
The Netherlands	2015w50–2016w7	Date	1/5	10/12	94.8	6.9 to 99.7	–
Portugal	2015w51–2016w8	Date, cancer, obesity	3/14	14/35	11.9	−372.7 to 83.6	–
Romania	2016w3–2016w14	Date, cancer, renal disease	4/58	6/100	-22.6	−490.3 to 74.6	–
Spain	2016w1–2016w14	Date, age, heart disease, dependency	63/100	151/206	22.5	−39.6 to 56.9	–
two-stage pooled	–	–	–	–	49.0	13.5 to 70.0	36.2%

CI: confidence interval; I MOVE+: Integrated Monitoring of Vaccines in Europe plus; IVE: influenza vaccine effectiveness; w: week (International Organisation for Standardisation (ISO) week).

^a^ Poland and Hungary were excluded from the two-stage analyses because there were no vaccinated controls in Poland and no cases in Hungary.

^b^ Date of symptom onset and age modelled as restricted cubic spline with four knots.

### Vaccine effectiveness against hospitalised influenza B

We included in this analysis 110 cases of influenza B, of whom 50 (46%) were vaccinated and 1,015 controls, of whom 588 (58%) vaccinated. The median age of cases and controls were 76 (IQR: 12 years) and 78 years (IQR: 12 years) respectively (p = 0.056). A lower proportion of cases than controls had cancer (18% vs 28%, p = 0.037), a functional impairment (18% vs 36%, p < 0.001), and had been hospitalised in the previous 12 months (36% vs 48%, p = 0.02). The proportion of current smokers was higher among influenza B cases than among controls (35% vs 23%, p = 0.007) (Table 2).

One stage pooled IVE against influenza B adjusted for symptom onset time and age was 52% (95% CI: 24 to 70) and 47% (95% CI: 13 to 68) when further adjusted for a range of underlying conditions and hospitalisation in the previous year (Table 5). IVE was 62% (95% CI: 5 to 85), 60% (95% CI: 18 to 80) and 36% (95% CI: −23 to 67) in patients with diabetes mellitus (n = 302), lung (n = 520) and heart disease (n = 675), respectively.

**Table 5:** Pooled adjusted seasonal vaccine effectiveness against hospitalised influenza B among elderly people overall and by risk groups, I-MOVE + study, Europe, influenza season 2015/16

	Model used for adjustment^a^	Vaccinated /cases	Vaccinated /controls	Adjusted IVE	95% CI
**Overall**					
Overall	Initial	50/110	588/1,015	51.8	23.7 to 69.5
Overall	Full	46/101	544/948	47.0	13.1 to 67.7
**By risk groups**					
At least one underlying condition	Initial	47/97	536/929	50.2	18.7 to 69.4
	Initial plus severity			49.4	17.5 to 69.0
Diabetes mellitus					
No	Initial	33/72	404/696	40.9	−7.1 to 67.4
Yes	Initial	13/30	152/272	62.1	5.8 to 84.7
	Initial plus severity			62.0	5.3 to 84.8
Heart disease					
No	Initial	16/44	211/368	66.5	27.6 to 84.5
Yes	Initial	32/61	354/614	36.3	−22.2 to 66.8
	Initial plus severity			36.1	−22.9 to 66.7
Lung disease					
No	Initial	27/61	258/502	32.8	−28.6 to 64.8
Yes	Initial	22/45	305/475	60.5	19.2 to 80.6
	Initial plus severity			59.9	18.2 to 80.4
**Vaccine type**					
Inactivated subunit	Initial	20/61	207/542	49.0	13.5 to 70.0
Inactivated split virion	Initial	30/74	332/652	54.1	18.9 to 74.0
**Sensitivity analyses**					
two-stage model	two-stage^b^	48/86	551/858	47.0	11.9 to 68.2
Restricted to patients swabbed within 3 days	Initial	31/54	358/585	25.0	−50.5 to 62.6
Restricted to patients not receiving antivirals before swabbing	Initial	46/99	577/985	52.3	22.8 to 70.5

CI: confidence interval; I MOVE+: Integrated Monitoring of Vaccines in Europe plus; IVE: influenza vaccine effectiveness.

^a^ Initial: one-stage model adjusted for study site, date of onset and age (modelled as restricted cubic splines). Full: one-stage model adjusted for study site, date of symptom onset, age (modelled as restricted cubic splines), lung, heart, renal disease, diabetes mellitus, cancer, obesity and hospitalisation in the previous year Severity: hospitalisations for underlying conditions in the previous year.

^b^ Croatia, Hungary, Italy, Lithuania, the Netherlands, Poland, Portugal and Romania were excluded from the two-stage analyses because there were no vaccinated controls and/or cases, respectively.

IVE against influenza B was 49% (95% CI: 14 to 70) for inactivated subunit vaccines (n = 603) and 54% (95% CI: 19 to 74) for inactivated split virion vaccines (n = 726).

Study site specific IVE ranged between 18% (95% CI: −106 to 67) in Finland (n = 38) and 76% (95% CI: −24 to 95) in Italy (n = 259) (Table 6). There was no statistical heterogeneity between study site specific IVE estimates (I2 = 0%). The two-stage pooled analysis (n = 944) included Finland, France, Italy, Navarre and Spain. IVE was 47% (95% CI: 12 to 68). In sensitivity analyses, IVE against influenza B was 52% (95% CI: 23 to 71) when restricting to patients not having received antiviral treatment (n = 1,084) and 25% (95%CI: −51 to 63) among patients swabbed within 3 days of symptoms onset (n = 639).

**Table 6:** Study site-specific and two-stage^a^ pooled seasonal vaccine effectiveness against hospitalised influenza B among elderly people, I- MOVE + study, Europe, influenza season 2015/16

Study site	Inclusion period	Variables used for adjustment^b^	Vaccinated /cases	Vaccinated /controls	Adjusted IVE	95% CI	I-square
Finland	2016w8–2016w15	Date	2/3	22/35	23.3	-1,785.9 to 96.9	–
France	2016w4–2016w14	Date, age, functional impairment	17/26	87/120	18.1	-105.6 to 67.4	–
Italy	2016w1–2016w12	Date	2/10	121/249	75.5	-23.7 to 95.1	–
Navarre	2015w53–2016w17	Date	17/27	165/230	59.4	-2.5 to 83.9	–
Spain	2016w2–2016w16	Date, lung disease, dependency	11/20	159/220	44.3	-48.9 to 79.1	–
two-stage pooled (n = 944)	–	–	–	–	47.0	11.9 to 68.2	0.0%

CI: confidence interval; I MOVE+: Integrated Monitoring of Vaccines in Europe plus; IVE: influenza vaccine effectiveness; w: week (International Organisation for Standardisation (ISO) week).

^a^ Croatia, Hungary, Italy, Lithuania, the Netherlands, Poland, Portugal and Romania were excluded from the two-stage analyses because there were no vaccinated controls and/or cases.

^b^ Date of onset and age modelled as restricted cubic spline with four knots.

## Discussion

Our results suggest that the seasonal IVE against hospitalised influenza among elderly people was moderate during the 2015/16 influenza season in Europe for influenza: 39% overall, 42% against influenza A(H1N1)pdm09 and 52% against influenza B . These estimates did not vary between categories of underlying conditions.

Data from European virological surveillance reported that most of the characterised influenza A(H1N1)pdm09 viruses belonged to the emerging subclade 6B.1, defined by haemagglutinin amino acid substitutions S162N and I216T [18]. Despite these genetic evolutions, A(H1N1)pdm09 viruses were considered antigenically similar to the northern hemisphere vaccine component A/California/7/2009. IVE estimates against hospitalised A(H1N1)pdm09 was consistent with the results we reported in 2012/13 and 2013/14 among hospitalised elderly people [24,25].

In 2015/16, the circulating influenza B Victoria lineage was distinct from the Yamagata vaccine component [26] and there was no quadrivalent vaccine used in our study population. IVE against influenza B was close to what we reported, using the same generic protocol, in 2012/13 (66% in the 65–79 year-olds and 44% in the 80 year-olds and older) in a season with co-circulation of B Victoria and Yamagata lineages and a Yamagata vaccine component [24,27]. These results suggest some cross-lineage protection and they are in line with previously reported data in GP-based studies [28,29] and a meta-analysis of eight randomised controlled trials with mismatched B viruses resulting in a VE of 52% (95% CI: 19 to 72) among healthy adults [30]. Further studies are needed to increase the understanding of mechanisms of cross-lineage protection for influenza B and better guide policy makers in terms of recommendations for using trivalent or tetravalent seasonal vaccines.

We observed higher point estimates of IVE for the inactivated split virion vaccines compared with inactivated subunit vaccines, although the 95% CIs of the point estimates were widely overlapping. The completeness of data on vaccine type was high (1% of missing vaccine type among those vaccinated), thus these results, concurring with published data [31-33], could be due to differences in T-cell responses conferred by the two vaccine types [34]. However, they should be interpreted with caution as they may be due to random variation. Further evidence, and pooling of several years of data would be required to obtain precise vaccine type specific effectiveness. Higher adjuvant vaccine coverage would be needed to compute adjuvant vaccine specific IVE. This would be useful information to adapt influenza vaccination strategies among elderly people.

Recent reviews underlined the need for further evidence of seasonal IVE against laboratory-confirmed influenza in elderly people and patients with underlying conditions [8-13]. We were able to collect high quality data from 1,802 elderly patients hospitalised with SARI, making our study one of the largest hospital-based studies on IVE. The large number of participants, and a vaccine coverage close to 50%, enabled us to compute IVE against type/subtype-specific influenza among patients with specific underlying conditions. Our results suggest that, in 2015/16, the seasonal influenza vaccine provided protection against hospitalised influenza A(H1N1)pdm09 and B in the elderly with diabetes mellitus, heart and lung disease. We were unable to refine the underlying conditions categories further. To better guide vaccine recommendations, IVE among patients receiving specific treatment (e.g. statins [35,36], chemotherapy [9,37]) or with more specific conditions (e.g. asthma, chronic obstructive pulmonary disease) would be needed. A larger sample size would be required for such studies.

We collected information related to access to care, health conditions and smoking status. Recruited cases and controls were similar. We adjusted our estimates for study site, onset week and age. Further adjustment for potential confounders (underlying lung, heart, renal disease, diabetes mellitus, cancer, obesity and hospitalisations in the past year) did not change the estimates. However, as in any observational study, we cannot exclude unmeasured confounding leading to over- or under-estimation of the IVE.

The contribution to the pooled dataset was different between study sites. The two Spanish sites recruited 44% of the patients. The viruses circulating and vaccines used in Spain were similar to the other countries. Consequently we do not expect the over-representation of Spanish sites to have biased our overall estimates. Variations in the number of recruited individuals may be explained by differences in local influenza activity or number and size of participating hospitals/services. We believe that access to hospitalisation in case of severe influenza is similar across participating European countries. A common generic protocol and the monitoring of its implementation through on-site visits contributed to ensuring comparability of patients recruited and data collected across study sites. We measured low statistical heterogeneity based on I-square values. However, small number of estimates and large study-specific CIs may hinder adequate quantitative assessment of heterogeneity between studies [38]. True differences between study site specific IVE could be related to different vaccines used during this season or different immunological profiles of recruited patients including their past vaccination histories [39]. Larger study site-specific sample sizes are required to ensure that the differences in IVE across study sites are not due to chance. Currently, multicentre studies are necessary to obtain precise IVE estimates.

A recent publication by Foppa et al. suggested that measuring IVE against laboratory-confirmed influenza SARI hospitalisation using the TND was subject to biases if the test-negative controls were hospitalised because of an exacerbation of underlying lung disease unrelated to a respiratory infection [40]. In our study, cases and controls had similar prevalence of underlying lung disease. Underlying lung disease did not appear to confound IVE estimates, even when combined with a proxy of its severity (hospitalisation because of underlying conditions in the past 12 months). Cohort and TND-based IVE estimates against laboratory-confirmed hospitalised influenza in Navarre repeatedly showed similar estimates, reassuring on the appropriateness of TND at hospital level [41].

Several studies suggest that past influenza vaccinations may decrease or enhance current vaccine effectiveness depending on previous and current vaccine and circulating strains as well as past exposure to the virus [24,32,42-44]. A large proportion of our vaccinated population had been vaccinated in the previous season(s) but the very small number of patients with varying repeated vaccination status over the years did not allow us to measure the effect of previous vaccinations. To understand the effect of repeated vaccinations on IVE, large cohorts of individuals with different vaccination patterns and symptomatic (and asymptomatic) influenza infection status over the years would be needed.

## Conclusion

Our multicentre test-negative case–control study estimated that in 2015/16 the seasonal influenza vaccination prevented approximately half of the cases of hospitalisation with laboratory-confirmed influenza among vaccinated elderly people. Our results suggest that vaccination provided similar protection to elderly patients with underlying diabetes mellitus, cancer, lung and heart diseases. Because vaccination remains the most effective preventive measure against severe influenza among elderly people, increasing the vaccine coverage in this group should be a priority. This pilot season of the hospital-based I-MOVE + project proved that obtaining precise estimates of IVE against a severe influenza outcome among elderly people was feasible. Enlarging our network and its sample size will enable us to better guide vaccination strategies against severe influenza cases by comparing the performance of different vaccine types and identifying risk groups for poor response to vaccination.

## Notes

**I-MOVE + hospital working group:** EpiConcept: Alain Moren Spain: Jone M. Altzibar, Ion García Arraras (Subdirección de Salud Pública de Gipuzkoa, CIBERESP, Basque Country, Spain), Gustavo Cilla (Donostia University Hospital, CIBERES, San Sebastian, Basque Country, Spain), Elisa Marco (Dirección General de Salud Pública, Aragón, Spain), Matxalen Vidal, Manuel Omeñaca (Miguel Servet University Hospital, Zaragoza, Spain) Navarra: J. Castilla (Instituto de Salud Pública de Navarra, IdiSNA, CIBERESP, Pamplona), A Navascués, C Ezpeleta (Complejo Hospitalario de Navarra, IdiSNA, Pamplona, Spain), L Barrado (Hospital de Estella, Spain), MT Ortega (Hospital de Tudela, Spain) Italy: A. Bella, M.R. Castrucci, S. Puzelli (Istituto Superiore di Sanità, Rome), M.Chironna, C. Germinario (Policlicnico Hospital, University of Bari); F. Ansaldi, A. Orsi (IRCCS AOU San Martino- IST, Genoa hospital); I. Manini, E. Montomoli (Department of Molecular and Developmental Medicine, University of Siena) Romania: E.Lupulescu, M.Lazar, CM Cherciu, C. Tecu, ME Mihai (National Institute of Research Cantacuzino), M. Nitescu (INBI Matei Bals, Bucharest), D. Leca (UMF Gr. Popa Iasi), E. Ceausu (UMF Carol Davila Bucharest, Infectious Diseases Hospital V. Babes, Bucharest) France: N. Lenzi, Z. Lesieur (Innovative clinical research network in vaccinology (I-REIVAC), P. Loulergue (Inserm, CIC Cochin-Pasteur, I-REIVAC, Paris), V. Foulongne, F. Letois, C. Merle (CHU de Montpellier, I-REIVAC), P. Vanhems (Hôpital Edouard Herriot, Lyon, I-REIVAC), B. Lina (Université Lyon 1, Centre National de Référence virus influenza France Sud, Lyon, France) Finland: H. Nohynek, A. Haveri (National Institute for Health and Welfare) Lithuania: M. Kuliese, D. Velyvyte (Department of Infectious Diseases of Lithuanian University of Health Sciences, Kaunas, Lithuania), R. Grimalauskaite, G. Damuleviciene, V. Lesauskaite (Department of Geriatrics, Lithuanian University of Health Sciences, Kaunas, Lithuania), L. Jancoriene, B. Zablockiene, A. Ambrozaitis (Clinic of Infectious, Chest Diseases, Dermatovenerology and Allergology, Vilnius University Faculty of Medicine, Vilnius, Lithuania) Portugal: B. Nunes, A.P. Rodrigues (National Health Institute Doutor Ricardo Jorge, Lisbon), V. Gomes, R. Côrte-Real (Centro Hospitalar de Lisboa Central, Lisbon), J. Poças, M.J. Peres (Centro Hospitalar de Setúbal, Setúbal) Croatia: B. Kaić (Croatian Institute of Public Health, Zagreb) Hungary: B. Oroszi (Office of the Chief Medical Officer, Budapest) Poland: L.B. Brydak, K. Cieślak, D. Kowalczyk, K. Szymański (National institute of Public Health - National Institute of Hygiene, Warsaw), A. Jakubik (Szpital Praski, Warsaw), G. Skolimowska (Mazovia Regional Hospital, Siedlce), D. Hulbój (Regional hospital, Bielsko-Biała) The Netherlands: A. Meijer, W. van der Hoek (National Institute for Public Health and the Environment (RIVM), Bilthoven), P.M. Schneeberger (Jeroen Bosch Hospital, 's Hertogenbosch)
